# Circadian Rhythms and Melatonin Metabolism in Patients With Disorders of Gut-Brain Interactions

**DOI:** 10.3389/fnins.2022.825246

**Published:** 2022-03-09

**Authors:** Sophie Fowler, Emily C. Hoedt, Nicholas J. Talley, Simon Keely, Grace L. Burns

**Affiliations:** ^1^School of Biomedical Sciences and Pharmacy, College of Health, Medicine and Wellbeing, The University of Newcastle, Newcastle, NSW, Australia; ^2^NHMRC Centre of Research Excellence in Digestive Health, The University of Newcastle, Newcastle, NSW, Australia; ^3^Hunter Medical Research Institute, New Lambton Heights, NSW, Australia; ^4^School of Medicine and Public Health, College of Health, Medicine and Wellbeing, The University of Newcastle, Newcastle, NSW, Australia

**Keywords:** functional gastrointestinal disorder, functional dyspepsia, disorders of gut brain interaction, melatonin, circadian rhythm, irritable bowel syndrome

## Abstract

Circadian rhythms are cyclic patterns of physiological, behavioural and molecular events that occur over a 24-h period. They are controlled by the suprachiasmatic nucleus (SCN), the brain’s master pacemaker which governs peripheral clocks and melatonin release. While circadian systems are endogenous, there are external factors that synchronise the SCN to the ambient environment including light/dark cycles, fasting/fed state, temperature and physical activity. Circadian rhythms also provide internal temporal organisation which ensures that any internal changes that take place are centrally coordinated. Melatonin synchronises peripheral clocks to the external time and circadian rhythms are regulated by gene expression to control physiological function. Synchronisation of the circadian system with the external environment is vital for the health and survival of an organism and as circadian rhythms play a pivotal role in regulating GI physiology, disruption may lead to gastrointestinal (GI) dysfunction. Disorders of gut-brain interactions (DGBIs), also known as functional gastrointestinal disorders (FGIDs), are a group of diseases where patients experience reoccurring gastrointestinal symptoms which cannot be explained by obvious structural abnormalities and include functional dyspepsia (FD) and irritable bowel syndrome (IBS). Food timing impacts on the production of melatonin and given the correlation between food intake and symptom onset reported by patients with DGBIs, chronodisruption may be a feature of these conditions. Recent advances in immunology implicate circadian rhythms in the regulation of immune responses, and DGBI patients report fatigue and disordered sleep, suggesting circadian disruption. Further, melatonin treatment has been demonstrated to improve symptom burden in IBS patients, however, the mechanisms underlying this efficacy are unclear. Given the influence of circadian rhythms on gastrointestinal physiology and the immune system, modulation of these rhythms may be a potential therapeutic option for reducing symptom burden in these patients.

## Introduction

Circadian rhythms are endogenously generated cycles of molecular, physiological and behavioural events that occur within an organism approximately every 24 h. These rhythms are created and maintained by independent transcriptional and translational feedback loops in peripheral tissues (known as peripheral clocks) which are controlled by the suprachiasmatic nucleus (SCN), the master pacemaker located in the hypothalamus (Plautz et al., 1997). The activity of these genes (including Circadian Locomotor Output Cycles Kaput, *CLOCK*; Brain and Muscle ARNT-Like 1, *BMAL1*; referred to as “clock genes”) drive circadian rhythms and can be synchronised to the ambient environment by external signals called zeitgebers (German for “time giver”) including light (Johnson et al., 1988), feeding times (Damiola, 2000), ambient temperature (Hastings and Sweeney, 1957), and physical activity (Edgar and Dement, 1991). Within the gastrointestinal tract (GIT), circadian cycles influence the immune system, microbiome and mucosal homeostasis (Rosselot et al., 2016).

Synchronisation of peripheral clocks is largely controlled by the hormone melatonin, the production of which is governed by the SCN and is entrained to the light/dark cycle. The melatonin cycle conveys information about daily light/dark cycles to synchronise peripheral clocks, therefore stabilising and coupling circadian rhythms (Redman et al., 1983). Dysregulation of these rhythms is associated with a number of metabolic and inflammatory diseases including obesity, metabolic dysfunction and disorders of gut-brain interactions (DGBIs) (Voigt et al., 2019).

Disorders of gut-brain interactions, including functional dyspepsia (FD) and irritable bowel syndrome (IBS), are a group of disorders with a strong association with sleep disturbances but with relatively little known about circadian involvement (Koh et al., 2014; Yamawaki et al., 2014). These patients experience reoccurring GI symptoms which cannot be explained by obvious structural abnormalities (Drossman, 1999). FD is a multifactorial DGBI primarily affecting the upper GIT and is defined as having one or more of the following symptoms: early satiety, postprandial fullness, or epigastric pain or burning, in the absence of structural disease (Futagami et al., 2018). These symptom profiles can be used to subtype patients into postprandial distress syndrome (PDS) which describes symptoms of postprandial fullness and early satiety, and epigastric pain syndrome (EPS) which incorporates epigastric pain and burning, however, there is significant overlap between these profiles (Stanghellini et al., 2016). IBS is characterised by abdominal pain associated with changes in stool consistency with no underlying evidence of organic disease and is subtyped based on stool consistency (Camilleri et al., 2012). Importantly, over 40% of the global population experience GI symptoms associated with these conditions (Sperber et al., 2021), highlighting the need to better understand the mechanisms underlying symptom onset and chronicity. Further, alterations in immune profiles, microbiota composition and physiology of the GIT are linked to DGBIs (Burns et al., 2021), all of which are associated with chronodisruption and as such, it is likely that altered circadian rhythms are involved in the pathophysiology of these conditions. This review aims to discuss the evidence for circadian abnormalities in DGBIs with a focus on FD and IBS.

## Regulation of the Central Circadian Clock

Light is the primary zeitgeber for the SCN and determines the phase of the circadian clock. The retina carries photic information via the retinohypothalamic tract to the SCN which will adjust the circadian phase (phase shift) based on the ambient light levels (Berson, 2002). Exposure to natural daylight at high intensities advances the circadian clock, affecting sleep duration, and improving sleep quality (Roenneberg et al., 2003; Boubekri et al., 2014). In contrast, light exposure at evening and night-time will delay the clock, leading to later sleep cues (Wright et al., 2013; Wams et al., 2017). A well reported example of this is the use of smartphones before bedtime, this has been shown to lead to decreased sleep quality, longer sleep onset latency and delays in sleep which shortens sleep duration (Lemola et al., 2015; Christensen et al., 2016).

Feeding/fasting patterns also cause phase shifts within the circadian system, and this may be of importance in diseases of the GIT. Sustained patterns of feeding/fasting can synchronise peripheral clocks and erratic eating patterns can disrupt this coordination. This has been demonstrated in mice, whereby restricting access to food during the inactive phase caused a phase shift in peripheral clocks (Damiola, 2000), as indicated by increased *PER2* and *BMAL1* mRNA expression in the stomach (Laermans et al., 2014). Late meals result in delayed Period 2 (*PER2*) mRNA rhythms in adipose tissue (Wehrens et al., 2017), and reduced calorie intake can also initiate a phase shift in peripheral clocks (Luna-Moreno et al., 2009; Arellanes-Licea et al., 2014). The nuclear receptor subfamily, Reverse Strand of Gene Encoding Thyroid Hormone Receptor (REV-ERB), is involved in rhythm generation in the circadian system and acts to regulate clock outputs in metabolic pathways (Bugge et al., 2012; Cho et al., 2012). This is seen in mice lacking *REV-ERB*α, who lose daily food intake rhythms, as well as in mice living in constant darkness (Sen et al., 2018), highlighting the impact of food intake in the regulation of circadian rhythms and demonstrates its importance in maintaining normal physiological functioning. This may to some extent explain why patients with FD report reduced symptom burden when altering their meal habits to include smaller portions and more regular feeding schedules (Duncanson et al., 2021). Importantly, modulation of feeding patterns may have utility as a clinical approach for re-synchronising circadian clocks.

### Transcription and Translational Control of Circadian Rhythms

**FIGURE 1:**
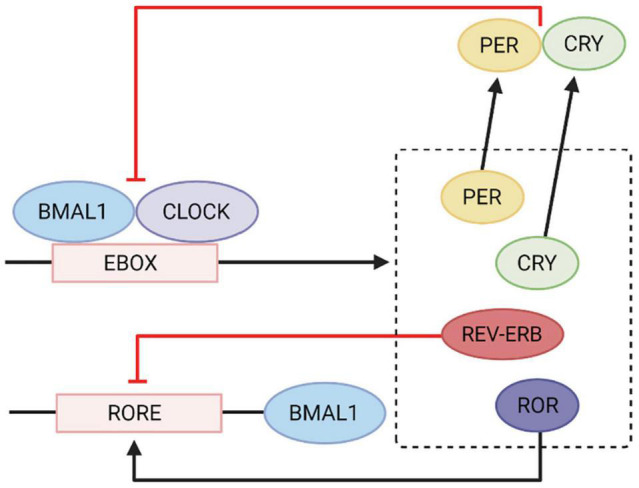
Circadian gene transcription and translational feedback loop. *CLOCK* and *BMAL1* genes heterodimerise and bind to the E-box element on *PER*, *CRY*, *ROR*, and *REV-ERBA* genes which activates transcription. *CRY* and *PER* gene products inhibit *CLOCK* and *BMAL1* gene expression. *REV-ERB* inhibits the RORE element on *BMAL1* genes and *ROR* activates it to increase *BMAL1* gene expression. Adapted from Wang et al. (2014) International Journal of Molecular Sciences. Image created using Biorender.com.

The negative limb of the feedback loop involves *CLOCK* and *BMAL1* genes, which form heteromeric complexes during the day to activate the transcription of *PER1*, *PER2*, *PER3*, Cryptochrome 1 (*CRY1*), *CRY2*, *REV-ERB* and retinoid-related orphan nuclear receptor (*ROR*) genes (Figure 1). After sufficient protein levels of nuclear *PER* and *CRY* are reached over the course of the day, *CLOCK* and *BMAL1* gene expression is then repressed at night. As *PER* and *CRY* protein production begins to decline in the early morning, the cycle continues (Shearman, 2000; Mrosovsky et al., 2001). The positive limb of the feedback loop involves *REV-ERB* decreasing *BMAL1* gene expression and *ROR* increasing *BMAL1* gene expression (Bae et al., 1998, 2000; Preitner et al., 2002). These gene expression changes occur in multiple organ systems, including the GIT where circadian rhythms influence the physiology of digestion and motility. Consequently, alterations in physiological function, such as changes in permeability or transit times, may result in dysregulation of the circadian cycle.

### Peripheral Circadian Rhythms in the Gastrointestinal Tract

Circadian rhythms are a main regulator of digestion and absorption of nutrients, epithelial barrier function, motility and microbial activity. For example, saliva flow rate and secretory levels are diurnal (Dawes, 1972) salivary glands express clock genes (Zheng et al., 2012), suggesting regulation by circadian cycles. In the stomach, gastric acid secretion (Moore and Halberg, 1986), and disaccharidase activity (Stevenson et al., 1980) also show diurnal fluctuation. Additionally, GIT motor patterns also show diurnal rhythmicity that is dependent on meal timing and body position (Goo et al., 1987; Chitkara et al., 2004; Grammaticos et al., 2015). Further evidence from murine and cell line studies show that circadian rhythms contribute to control of the expression of tight-junction proteins within the intestinal epithelial barrier. Research has shown that occludin and claudin 1 are regulated by the binding of *CLOCK-BMAL1* to E-boxes in their promotor regions and their expression shows diurnal fluctuations which is lost in *PER2* mutant mice (Oh-Oka et al., 2014), highlighting consideration of rhythmicity may be important in determining mechanisms associated with motility disorders in the GIT, including DGBIs.

There is also evidence that the GI microbiota is under circadian control and the composition, function and abundance of the microbiota show diurnal fluctuations (Thaiss et al., 2014; Zarrinpar et al., 2014; Liang et al., 2015). One example is that of *Klebsiella aerogenes* which expresses 24-h rhythms outside of the host and becomes more motile and swarms rhythmically in response to melatonin (Paulose et al., 2016). Another is the alteration in abundance of *Mucispirillum schaedleri* in the epithelial mucosa throughout light and dark periods (Thaiss et al., 2016). Additionally, *Synechococcus elongatus* has three clock genes (Kai A, B, and C), which oscillate in a 24-h rhythm, directing clock-regulated gene expression (Liu et al., 1995; Nakajima, 2005). Mutations in host clock genes have also been shown to affect the microbial composition (Liang et al., 2015; Thaiss et al., 2016), demonstrating intrinsic reliance on host rhythmic signals for homeostasis. There is mounting evidence to suggest that many functions within the GIT are controlled by the circadian system, and therefore disruptions to this system may be involved in disease and loss of homeostasis. Restoration of rhythmicity may be an effective management approach for several GI conditions, including DGBIs. One major limitation of this area of research is that these gene expression feedback loops have not been extensively studied within the GIT of humans in health or disease. However, a significant body of work has focussed on melatonin as a surrogate indicator of circadian synchronicity, given its regulation of peripheral and circadian clocks.

## Melatonin

**FIGURE 2:**
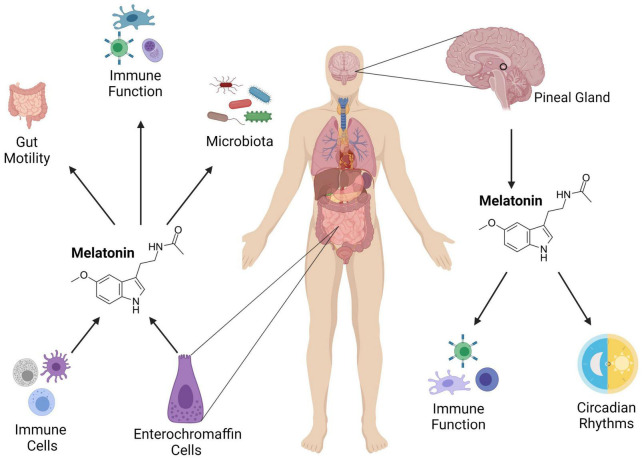
The role of melatonin in the intestinal immune system. Melatonin is produced from the pineal gland, and it influences circadian rhythms and immune function. It is also secreted in the gut from enterochromaffin and immune cells where it regulates GIT motility, immunity and the microbiota. Adapted from Ma et al. (2020) Medicinal Research Reviews. Image created using Biorender.com.

Given its importance in maintaining peripheral clocks, one potential avenue for restoring circadian rhythmicity may be supplementation with melatonin. In the GIT, melatonin is produced by enterochromaffin cells in the mucosa (Raikhlin and Kvetnoy, 1976) and aids in regulating both the immune system (Lardone et al., 2010) and intestinal motility (Figure 2; Harlow and Weekley, 1986; Storr et al., 2000). The concentration of melatonin in the GIT is suggested to be up to 400 times higher than in the pineal gland (Huether, 1994) and while pineal melatonin can enter the GIT through the circulatory system, GIT levels are independent of pineal levels (Bubenik and Brown, 1997), suggesting that melatonin plays a specific role in GI homeostasis.

There are two G-protein coupled melatonin membrane receptors, melatonin-1 receptor (MT1) and MT2. These receptors are found throughout the gut but are more localised to the large intestine and are involved in regulating motility, pain and signal transduction (Stebelová et al., 2010; Söderquist et al., 2015). There have been suggestions of an MT3 receptor capable of binding melatonin, however, sequencing has reclassified this as the quinone reductase two enzyme (Nosjean et al., 2000). The function of this enzyme is not fully understood but is hypothesised to transform quinone substrates into highly reactive compounds that can cause cellular damage and increase levels of reactive oxygen species (Calamini et al., 2008). Functionally, melatonin is believed to inhibit this enzyme which would explain its antioxidant and anti-inflammatory effects (Sharma and Haldar, 2006; Túnez et al., 2007). Due to the location of melatonin receptors throughout the GIT and their involvement in signal transduction and oxidation, decreased melatonin levels may participate in inflammation and disease processes within the GIT.

### Melatonin and Gastrointestinal Motility

Melatonin is involved in regulating GI motility through its dose-dependent excitatory and inhibitory effects on GI smooth muscle (Harlow and Weekley, 1986; Merle et al., 2000). Small doses of melatonin drive accelerated transit in the intestine and high doses reverse this effect, suggesting therapeutic capacity for melatonin in improving motor abnormalities frequently associated with DGBIs. Melatonin also decreases the force of spontaneous contractions in the ileum and colon, with one study demonstrating that melatonin can reduce the inhibitory non-adrenergic, non-cholinergic response in the GIT of rodents (Storr et al., 2002). This may be due to the reduced smooth-muscle inhibitory junction potential and inhibition of nitric oxide synthase activity at enteric synapses, suggesting that the site of melatonin action may potentially be neuronal rather than smooth muscle. This is an important consideration in the context of DGBIs, where proximity of nerve fibres to effector cells such as mast cells has been associated with abdominal pain (Barbara et al., 2004). These findings suggest that melatonin dosage affects GI motility and can impact on gastric emptying, however, the exact mechanism remains unclear and the therapeutic utility of specific melatonin dosages in improving motility abnormalities warrants further investigation.

### Melatonin and Intestinal Microbial Metabolism

Melatonin levels affect both the microbes and production of microbial metabolites including biotin, butyrate, proline and propionate (Liang and Fitzgerald, 2017) which are involved in GIT homeostasis and regulation of the immune system. In addition, the microbiota is also important in modulating tryptophan metabolism, an essential amino acid that contributes to normal growth and health. Tryptophan is the precursor to serotonin and melatonin and has immunomodulatory properties (Höglund et al., 2019) that affect gastric emptying (Geeraerts et al., 2011) and barrier function (Scott et al., 2020). Studies have shown that the microbiota can directly regulate peripheral tryptophan levels by stimulating serotonin production from enterochromaffin cells (Yano et al., 2015). However, over 90% of tryptophan is oxidised into kynurenine via the kynurenine pathway by the microbiota (Badawy, 2017), and this metabolite has been linked to inflammation (Nelp et al., 2019). Additionally, increased kynurenine levels have been associated with depressive-like behaviours (O’Connor et al., 2009). In the large intestine, commensal microbes, including *C. sporogenes* and *R. gnavus*, also work to convert tryptophan into tryptamine (Williams et al., 2014), an indolamine metabolite. Tryptophan can also be metabolised into indole by *E. coli*, *Clostridium* and *Bacteroides* species (Lee and Lee, 2010), which leads to the production of nicotinamide adenine dinucleotide, a coenzyme that is integral for host metabolism (Kotake and Masayama, 1936). Indole has a protective capacity in the GIT by stimulating the production of glucagon-like peptide 1 (GLP-1) from enteroendocrine L cells (Natividad et al., 2018). Given the tryptophan-serotonin metabolic pathway is impaired in patients with DGBIs (Gao et al., 2020), modulation of this pathway may improve symptoms in these conditions.

### Melatonin and the Immune System

Melatonin may also be required for immunomodulation, given the enzymes and receptors required for melatonin synthesis are widely found on immune cells. For example, both N-Acetyltransferase and Hydroxyindole-O-Methyltransferase are required for melatonin synthesis and are produced in T-cells (Carrillo-Vico et al., 2004). Subsequent melatonin synthesised from T-cells is believed to be involved in regulating IL-2/IL-2R expression (Lardone et al., 2010), a key player in regulating T-cell proliferation (Willerford et al., 1995). There is also suggestion that melatonin may regulate differentiation of T helper (Th) response pathways. Melatonin has been suggested to promote Th1 mediated responses by up-regulating IL-2 and IFN-γ production by CD4^+^ cells (Garcia-Mauriño et al., 1997) and it was shown that melatonin increases the production of IFN-γ by murine splenocytes (Colombo et al., 1992). In contrast, another study found that melatonin increased IL-4 secretion, in conjunction with downregulation of IL-2 and IFN-γ, suggesting a T helper 2 response (Shaji et al., 1998). In addition, the chronic administration of melatonin in mice increased the production of IL-10 and decreased TNF-a which further highlights a skew toward a T helper 2 response (Raghavendra et al., 2001). While the literature implicates melatonin in enhancing the T cell response, these conflicting results may suggest melatonin is not associated with a specific T cell subset, but rather is involved in modulating the T cell response with the aim of restoring GI homeostasis. Alternatively, there may be a dose dependent response to melatonin that skews toward a Th1 or Th2 response.

Melatonin also enhances antigen presentation through increasing MHC class II expression and the production of IL-1 and TNF-α (Pioli et al., 1993). These findings were supported by investigation of the proteome of shift workers with circadian misalignment, which showed that proteins in multiple pathways linked to antigen presentation and interferon signalling were abnormal (Depner et al., 2018), suggesting an altered adaptive immune responses in relation to daily activities. A further study evaluating circulating white blood cells and the association with sleep patterns (Hoopes et al., 2021) found that irregular sleep patterns are associated with an increased total white blood cell count and circulating neutrophils, lymphocytes and monocytes. This may be associated with the immunomodulatory capacity of melatonin to regulate natural killer cells (Lewiñski et al., 1989), neutrophils (García-Mauriño et al., 1999; Peña et al., 2007) and macrophage (Barjavel et al., 1998) responses. Further, melatonin inhibits neutrophil accumulation during inflammation, preserving mucosal cell integrity through reduction of neutrophil-mediated damage (Ercan et al., 2004), suggesting alterations in melatonin release associated with irregular sleep patterns may contribute to inflammation. In animal models of dextran sodium sulphate-induced colitis, 7 weeks of daily intraperitoneal melatonin administration reduced the severity of intestinal inflammation (Pentney and Bubenik, 1995). However, while acute melatonin treatment limits acute inflammatory damage, treatment is deleterious to chronic models of colitis (Marquez et al., 2006). This may be because acute administration of melatonin reduced the activity of myeloperoxidase, an enzyme that increases reactive oxidant species (Pattison et al., 2012), while the harmful impact of chronic melatonin administration may be due to its immunostimulatory effects. As such, the dosage of melatonin likely determines the impact on the immune system, however, it is clear that melatonin plays an important role in regulating circadian rhythms, the immune system and gastrointestinal physiology. Therefore, dysregulation of this hormone may contribute to disease presentation.

### Melatonin and Sex Steroid Hormones

While there is no consensus regarding the specific differences and mechanisms at play, it is clear that sex differences exist in circadian rhythms and melatonin metabolism (Cipolla-Neto et al., 2021) which may in part explain the higher prevalence of DGBIs in women (Burns et al., 2021). In healthy women, the intrinsic circadian period of melatonin and body temperature are shorter (Duffy et al., 2011) and females also tend to wake up earlier, with morning chronotypes (Adan and Natale, 2002; Cain et al., 2010). The circadian patterns of glucocorticoid hormone secretion and clock gene expression also display sex differences, with testosterone suggested to modulate rhythmicity in the adrenal glands of mice (Kloehn et al., 2016) and the secretion of glucocorticoid hormone conveys key timing information to both the central and peripheral circadian system (Balsalobre et al., 2000; Lamia et al., 2011). As such, the effects of sex hormones on adrenal gland rhythmicity may link to the dysregulation of stress hormones, such as corticotropic releasing hormone in DGBIs (Tanaka et al., 2016; Kano et al., 2017) but this relationship remains largely unexplored.

Melatonin plays an important role in regulating sex steroid hormones and influences the synthesis and release of gonadotropin releasing hormone (Bittman et al., 1985). For example, a study in males with gonadotropin releasing hormone deficiencies demonstrate that treatment with testosterone decreased melatonin levels back to within normal ranges (Luboshitzky et al., 1996). While the influence of melatonin on male sex steroid hormones is not fully understood, it helps regulate testosterone secretion and influences the growth, proliferation and secretory activity of testicular cells (Frungieri et al., 2017). Melatonin is also involved in multiple pathways for regulating the physiology of Sertoli cells (Yang et al., 2014) and influencing spermatogenesis (Espino et al., 2011; Moayeri et al., 2018). In terms of female reproductive hormones, there is evidence that the oral contraceptive pill increases melatonin levels (Boivin et al., 2016). Melatonin synthesis occurs in the ovary (Itoh et al., 1997), oocytes (Sakaguchi et al., 2013) and placenta (Iwasaki et al., 2005); and the ovaries have been shown to express the MT1 and MT2 receptors (Niles et al., 1999; Woo et al., 2001), however, the specific implications of melatonin production and metabolism in the female reproductive tract are not fully understood. While there are a number of sex differences with regard to circadian cycles and melatonin levels, there are considerable inconsistencies reported between studies (Cipolla-Neto et al., 2021), likely due to confounding factors such as the contraceptive pill (Kostoglou-Athanassiou et al., 1998) and age (Fernandez et al., 1990), making it hard to draw firm conclusions about the biological consequences of such differences. However, it appears the contribution of sex to circadian rhythmicity may link to the sex differences reported in DGBI prevalence and physiology.

### Mechanisms of Chronodisruption

Studies highlight a relationship between sleep disturbances, mental health and GI dysmotility (Nojkov et al., 2010; Kim et al., 2013; Saeed and Galal, 2017), linking chronodisruption to altered GI function. This is further supported by the observation that those with chronic insomnia have a higher prevalence of GI problems (Taylor et al., 2007; Bhaskar et al., 2016; Hyun et al., 2019). While the mechanisms underpinning these observations are not fully understood, polymorphisms in *PER3* are associated with increased susceptibility to Crohn’s disease (CD) and a more severe symptom profile (Mazzoccoli et al., 2012). In inflammatory diseases, including CD, disrupted circadian rhythms can both provoke and worsen the inflammation (Preuss et al., 2008; Tang et al., 2009). Further, polymorphisms in *CLOCK* and *PER* genes are associated with decreased gastric motility in the morning (Yamaguchi et al., 2015) and animal studies have demonstrated that *PER1/2* knockout mice have decreased colonic motility (Hoogerwerf et al., 2010), suggesting that dysregulation of circadian rhythm drives GI symptoms. In addition, mice with deficient *PER1/2* genes had decreased GI barrier integrity due to impaired cell division (Pagel et al., 2017). Importantly, under inflammatory conditions, there was a loss of Paneth and goblet cells and a decrease in transcription of barrier protective genes, indicating reduced barrier protection in Per1/2 mice (Pagel et al., 2017), highlighting that regulated circadian rhythms are essential for mucosal barrier integrity. In addition, feeding rhythms were disrupted in *CLOCK* mutant mice and restricted feeding recovered the appropriate rhythm (Yoshida et al., 2015), highlighting the importance of food intake when considering disruption to the circadian system. These studies suggest that alterations to circadian rhythms have a major impact on GI physiology and inflammatory pathways and may cause decreased barrier integrity. Further, these studies highlight the potential for restoring circadian rhythms in reducing disease flares and burden of GI disease on patients. One approach for restoring normal rhythm appears to be through control of feeding times. Normally, ghrelin secretion increases before a meal and declines postprandially (Cummings et al., 2001). However, this suppression of postprandial ghrelin is blunted in night workers (Schiavo-Cardozo et al., 2013), where feeding patterns are altered, highlighting circadian systems regulate hormone secretion. Night shift participants also had greater body fat mass percentage, lower insulin sensitivity and higher triglyceride levels compared to dayworkers (Schiavo-Cardozo et al., 2013), indicating increased adiposity and metabolic alterations. There is also evidence that chronodisruption influences GIT inflammation. Following an intermittent fasting schedule where participants fasted for 14–15 h each day for 25–30 days, resulted in decreased pro-inflammatory cytokines (Faris et al., 2012). Further, short-term fasting reduced circulating monocytes and markers of inflammation (Jordan et al., 2019), highlighting the importance of food intake timing in the regulation of GIT hormone secretion and immune function. These studies provide evidence that restricted feeding may realign the circadian system and help with symptom management, although this has not yet been extensively explored in GI disease cohorts.

Chronodisruption from altered light-dark cycles also leads to increased intestinal permeability (Summa et al., 2013) and alterations in the GIT microbiome (Wei et al., 1975; Kim et al., 2019). Shifts in the light/dark cycle causing chronic circadian misalignment resulted in a loss of colonic barrier function in mice (Tran et al., 2021). Sleep deprived mice have lower melatonin concentrations in colonic tissue in addition to increased colonic abundance in *Erysipelotrichales* and *Enterobacteriales*, which may induce inflammation, and decreased *Lactobacillales*, which is a beneficial bacterium in the GIT (Park et al., 2020). A further study (Deaver et al., 2018) found mice previously exposed to abnormal light-dark cycles demonstrated an altered GIT microbiota with increased abundance of *Ruminococcus torques*, associated with decreased intestinal barrier integrity, and subsequent loss of *Lactobacillus johnsonii*, a bacterial species which helps maintain the GIT epithelial cell layer. The increase in *Ruminococcaceae* spp. and decrease in *Lactobacillaceae* spp. is a common microbial profile seen in DGBI patients (Kassinen et al., 2007; Lyra et al., 2009; Rajiliæ-Stojanoviæ et al., 2011; Saulnier et al., 2011; Wang et al., 2013), suggesting these species may be associated with decreased integrity of the mucosal barrier. Further, there was a down regulation of genes involved in regulating important metabolic processes including 3-hydroxybutyrl-CoA dehydrogenase, acetaldehyde dehydrogenase, LPS export system permease protein and 3-deoxy-manno-octulosonate cytidylyltransferase (Deaver et al., 2018). These four genes are linked to tryptophan metabolism (Edenberg, 2007; O’Connor et al., 2009; Trefely et al., 2020) and downregulation of gene expression would therefore affect tryptophan metabolism, likely impacting on the availability of downstream products including serotonin and melatonin. These findings demonstrate that a loss of microbial richness may be involved in decreased GIT barrier integrity, mucous degradation (Hoskins et al., 1985), and immunomodulation (Pridmore et al., 2004). Moreover, decreased plasma melatonin levels and altered cytokine secretion has been demonstrated to cause mucosal injury in sleep deprived mice (Gao et al., 2019). There was an increase in catalase, superoxide dismutase and glutathione peroxidases which are antioxidant enzymes, associated with having decreased activity in IBS patients (Gurel, 2013). There was also upregulation of the proinflammatory cytokines IL-1, IL-6 and TNF-α, and the autophagic proteins, ATG5 and Beclin1. A concurrent downregulation of PCNA indicated increased cell autophagy and decreased cell proliferation (Gurel, 2013). Collectively, the immune profiles and the loss of microbial richness implicate chronodisruption with decreased enterocyte renewal and tight junctional protein levels and decreased integrity of the mucous layer. These studies highlight sleep deprivation can promote intestinal dysbiosis and aggravate GI disease and subsequently, improved sleep quality may help with symptom management. Further, given that supplementation with melatonin improved mucosal integrity in animal models of sleep deprivation (Gao et al., 2019, 2021), the therapeutic benefit of melatonin may be linked to strengthening of the intestinal barrier to reduce contact of luminal antigens with the mucosa.

## Disorders of Gut-Brain Interactions

Despite not being associated with mortality, DBGIs represent the most common diagnosis in gastroenterology, with over one-third of new patient consultations in secondary care gastroenterology clinics resulting in diagnosis of an DGBI (Shivaji and Ford, 2014). These patients are likely to account for more absenteeism from work, as well as reporting reduced productivity (Ballou et al., 2019), largely due to a lack of effective treatments for the conditions. Melatonin has been proposed as a potential therapy for IBS (Lu et al., 2005), however, specific mechanisms by which supplementation may improve the symptoms of DGBIs remain largely unknown. There is a strong correlation between DGBIs and sleep disturbances (Yamawaki et al., 2014; Yang et al., 2020; Koloski et al., 2021), indirectly indicating that circadian systems may be involved in their pathogenesis. Further, lifestyle factors including shift work have been associated with DGBI diagnosis (Knutsson and Bøggild, 2010; Nojkov et al., 2010; Kim et al., 2013), further supporting the hypothesis that dysregulated circadian cycles contribute to symptom chronicity in these patients. In addition, IBS patients reporting sleep disturbances were more likely to have higher symptom severity scores (Tu et al., 2017), linking circadian disruption to symptom manifestation. Rotating shift work is associated with increased psychological stress, poor quality sleep and greater experience of fatigue (Kim et al., 2013) which are all thought to contribute to a greater risk of chronic disease including cardiovascular disease and metabolic syndromes (Wang et al., 2011), demonstrating that an interruption to biological rhythmicity has significant impact on physiological function. This is also true in the GIT, where individuals performing rotating shift work and recovering from jet lag report increased incidences of GI symptoms including abdominal pain, constipation and diarrhoea (Caruso et al., 2004; Knutsson and Bøggild, 2010).

Acute tryptophan depletion is a method that reduces the availability of tryptophan in the brain and plasma, resulting in decreased serotonin and melatonin (Moja et al., 1988, 1989). As the microbiota metabolises dietary tryptophan the same decrease in serotonin and melatonin is likely reflected in the GIT. A study using this method on IBS patients (Kilkens, 2004) reported increased pain and urge scores as well as a lower perceptual threshold for first perception compared to placebo groups. As tryptophan is a precursor to melatonin, this suggests that melatonin may also be involved in visceral hypersensitivity and perception of pain. Additionally, tryptophan metabolite levels were significantly different between IBS and controls (Burr et al., 2019), demonstrated by a decrease in ten tryptophan metabolites (including indole-3-lactic acid and kynurenine) which were associated with sleep timing, hypothalamic-pituitary-axis hormones and (Burr et al., 2019), consistent with GIT melatonin influencing sleep and GI functioning that is altered in IBS.

With regards to FD, sleep disturbance is also more commonly reported in these patients compared to controls (Park et al., 2021). Further, symptom onset for a majority of patients is linked to eating and consequently some patients respond well to smaller, more regular meals (Duncanson et al., 2021). Interestingly, GI melatonin production is seemingly regulated by eating and food composition (Bubenik et al., 1996) rather than light/dark signals, suggesting abnormal melatonin production may be linked to FD symptom manifestation. In addition, serum and urinary melatonin concentrations are higher in patients with PDS compared to those with EPS and controls and there was a relationship between more severe symptoms and higher melatonin concentration (Chojnacki, 2011), suggesting chronodisruption in FD may result from abnormal melatonin production which inhibits regulation of the peripheral rhythms. This hypothesis can also be linked to the reported alterations in the FD microbiota, as small intestine biopsies from patients show a correlation between bacterial density in the mucosa of the small intestine and intensity of symptoms seen with food intake (Zhong et al., 2017). The low-grade mucosal inflammation, characterised by increased duodenal eosinophilia and mast cells in FD (Vanheel et al., 2014), and increased colonic mast cells in IBS (Walker et al., 2009) may also be the result of circadian disruption, however, this is largely unexplored so far.

### Therapeutic Evidence for Melatonin in Disorders of Gut-Brain Interactions

Melatonin has been investigated as a therapeutic agent for FD and IBS, however, the efficacy remains unclear. A study on paediatric FD found that melatonin had no significant effect (Zybach, 2016), however, another study found that FD participants showed significant improvement in sleep quality and abdominal pain (Klupinska et al., 2007). Similarly, disturbances in melatonin metabolism and secretion have been suggested to play a role in the pathophysiology of IBS (Radwan et al., 2009). A number of studies have examined the therapeutic effects of melatonin on patients with IBS in multiple clinical trials (Table 1) and the results show improved symptoms, sleep and mental health scores with reduced abdominal pain compared to placebo groups (Lu et al., 2005; Song, 2005; Saha et al., 2007; Chojnacki et al., 2013), however, no clear mechanism for these improvements has been identified. One hypothesis for this is that IBS patients have increased enterochromaffin cells (Lee et al., 2008), suggesting melatonin production may be altered and therefore supplementation mitigates this to reduce symptom burden. Further, increased melatonin and aralkylamine *N*-acetyltransferase (an enzyme involved in the production of melatonin) concentrations were seen in the colonic mucosa of patients with IBS-diarrhoea (Lee et al., 2008; Chojnacki, 2013). Melatonin can also be metabolised to 6-hydroxymelatonin sulphate which is excreted in the urine and one study reported patients with IBS having increased levels of this metabolite (Wisniewska-Jarosinska et al., 2010). As melatonin inhibits the activation and proliferation of mast cells (Izzo, 2004; Maldonado et al., 2016), it suggests that increasing melatonin availability may suppress the inflammatory profiles seen in IBS, however, there is little work on the specific mechanisms by which this may occur.

**TABLE 1:** Randomised clinical trials looking at efficacy of melatonin in patients with IBS.

References	Subjects	Treatment	Outcome
Lu et al., 2005	(Originally 24 in total) 17 females (10 in Group A and 7 in Group B), diagnosis based on Rome II	3 mg of melatonin or placebo every night for 8-weeks, 4-week washout period and then placebo or melatonin reversed	Improved bowel symptoms in IBS patients.
Saha et al., 2007	18 participants, 6 females (9 melatonin, 9 placebo), diagnosis based on Rome II	3 mg of melatonin or placebo at bedtime for 8 weeks.	Improved IBS and quality of life score, lower extracolonic IBS score.
Song, 2005	40 participants, 24 females (20 melatonin, 20 placebo), IBS diagnosis based on Rome II criteria, global Pittsburgh sleep quality index score of greater than 5	3 mg of melatonin or placebo for 2 weeks	Improved abdominal pain scores.
Chojnacki et al., 2013	80 postmenopausal females, IBS diagnosis based on Rome III, subtyped into IBS-C and IBS-D	3 mg of melatonin fasting and 5 mg at bedtime or placebo for 6 months	Decreased visceral pain, abdominal bloating and constipation.

## Conclusion

Disorders of gut-brain interactions affect a significant proportion of the global population and a lack of effective therapeutics for these conditions highlight the need for further research in the mechanisms of these conditions. Melatonin is involved in the regulation of GIT motility and sensation and evidence suggests an increased enterochromaffin cell number in patients with DGBIs. Furthermore, melatonin receptors, MT1 and MT2, are associated with the circadian regulation of immune cells and enhanced immune responses in the GIT, however, these mechanisms are not well explored in DGBIs.

Sleep disturbances, shift work and irregular eating patterns have all been linked to increased incidence of DGBIs implicating involvement of the circadian system in the pathophysiology. GIT barrier function, motility and immunity are heavily influenced by the circadian system and are important for gut-brain signalling. Given that circadian patterns regulate physiology, the immune system and the microbiota in the GIT, the therapeutic targeting of circadian patterns may reduce immune activation in DGBI patients.

## Author Contributions

SF performed the literature review and wrote the original draft of the manuscript. EH, NT, SK, and GB reviewed and edited the manuscript. All authors approved the final version of the manuscript submitted for publication.

## Conflict of Interest

NT non-financial support from HVN National Science Challenge NZ, personal fees from Aviro Health (Digestive health) (2019), Anatara Lifesciences, Brisbane (2019), Allakos (gastric eosinophilic disease) (2021), Bayer (2020), Danone (Probiotic) (2018), Planet Innovation (Gas capsule IBS) (2020), Takeda, Japan (gastroparesis) (2019), twoXAR (2019) (IBS drugs), Viscera Labs, (United States 2021) (IBS-diarrhoea), Dr. Falk Pharma (2020) (EoE), Censa, Wellesley, MA, United States (2019) (Diabetic gastroparesis), Cadila Pharmaceuticals (CME) (2019), Progenity Inc., San Diego, (United States 2019) (Intestinal capsule), Sanofi-Aventis, Sydney (2019) (Probiotic), Glutagen (2020) (Celiac disease), ARENA Pharmaceuticals (2019) (Abdominal pain), IsoThrive (2021) (oesophageal microbiome), BluMaiden (2021), Rose Pharma (2021), Intrinsic Medicine (2021) outside the submitted work; In addition, NT has a patent Nepean Dyspepsia Index (NDI) 1998, Biomarkers of IBS licensed, a patent Licensing Questionnaires Talley Bowel Disease Questionnaire licensed to Mayo/Talley, a patent Nestec European Patent licensed, and a patent Singapore Provisional Patent “Microbiota Modulation Of BDNF Tissue Repair Pathway” issued. Committees: Australian Medical Council (AMC) Council Member; Australian Telehealth Integration Program; MBS Review Taskforce; NHMRC Principal Committee (Research Committee) Asia Pacific Association of Medical Journal Editors. Boards: GESA Board Member, Sax Institute, Committees of the Presidents of Medical Colleges. Community group: Advisory Board, IFFGD (International Foundation for Functional GI Disorders). Miscellaneous: Avant Foundation (judging of research grants). Editorial: Medical Journal of Australia (Editor in Chief), Up to Date (Section Editor), Precision and Future Medicine, Sungkyunkwan University School of Medicine, South Korea, Med (Journal of Cell Press). NT is supported by funding from the National Health and Medical Research Council (NHMRC) to the Centre for Research Excellence in Digestive Health and he holds an NHMRC Investigator grant. SK Grant/Research Support: National Health and Medical Research Council (Ideas Grant and Centre for Research Excellence) Viscera Labs (Research contract), Microba Life Science (Research contract). Consultant/Advisory Boards: Gossamer Bio (Scientific Advisory Board), Anatara Lifesciences (Scientific Advisory Board), Microba Life Science (Consultancy). The remaining authors declare that the research was conducted in the absence of any commercial or financial relationships that could be construed as a potential conflict of interest.

## Publisher’s Note

All claims expressed in this article are solely those of the authors and do not necessarily represent those of their affiliated organizations, or those of the publisher, the editors and the reviewers. Any product that may be evaluated in this article, or claim that may be made by its manufacturer, is not guaranteed or endorsed by the publisher.

## References

[B1] Adan A. Natale V. (2002). Gender differences in morningness–eveningness preference. *Chronobiol. Int.* 19 709–720. 10.1081/cbi-120005390 12182498

[B2] Arellanes-Licea E. D. C. Báez-Ruiz A. Carranza M. E. Arámburo C. Luna M. Díaz-Muñoz M. (2014). Daily patterns and adaptation of the ghrelin, growth hormone and insulin-like growth factor-1 system under daytime food synchronisation in rats. *J. Neuroendocrinol.* 26 282–295. 10.1111/jne.12145 24617825

[B3] Badawy A. A. B. (2017). Kynurenine pathway of tryptophan metabolism: regulatory and functional aspects. *Int. J. Tryptophan Res.* 10:117864691769193. 10.1177/1178646917691938 28469468 PMC5398323

[B4] Bae K. Lee C. Hardin P. E. Edery I. (2000). dCLOCK is present in limiting amounts and likely mediates daily interactions between the dCLOCK–CYC transcription factor and the PER–TIM complex. *J. Neurosci.* 20 1746–1753. 10.1523/JNEUROSCI.20-05-01746.2000 10684876 PMC6772911

[B5] Bae K. Lee C. Sidote D. Chuang K.-Y. Edery I. (1998). Circadian regulation of a drosophila homolog of the mammalian clock gene: PER and TIM function as positive regulators. *Mol. Cell. Biol.* 18 6142–6151. 10.1128/MCB.18.10.6142 9742131 PMC109200

[B6] Ballou S. McMahon C. Lee H. N. Katon J. Shin A. Rangan V. (2019). Effects of irritable bowel syndrome on daily activities vary among subtypes based on results from the IBS in America Survey. *Clin. Gastroenterol. Hepatol.* 17:2471. 10.1016/j.cgh.2019.08.016 31419572 PMC7675784

[B7] Balsalobre A. Brown S. A. Marcacci L. Tronche F. Kellendonk C. Reichardt H. M. (2000). Resetting of circadian time in peripheral tissues by glucocorticoid signaling. *Science* 289 2344–2347. 10.1126/science.289.5488.2344 11009419

[B8] Barbara G. Stanghellini V. De Giorgio R. Cremon C. Cottrell G. S. Santini D. (2004). Activated mast cells in proximity to colonic nerves correlate with abdominal pain in irritable bowel syndrome. *Gastroenterology* 126 693–702. 10.1053/j.gastro.2003.11.055 14988823

[B9] Barjavel M. J. Mamdouh Z. Raghbate N. Bakouche O. (1998). Differential expression of the melatonin receptor in human monocytes. *J. Immunol.* 160 1191–1197. 9570533

[B10] Berson D. M. (2002). Phototransduction by retinal ganglion cells that set the circadian clock. *Science* 295 1070–1073. 10.1126/science.1067262 11834835

[B11] Bhaskar S. Hemavathy D. Prasad S. (2016). Prevalence of chronic insomnia in adult patients and its correlation with medical comorbidities. *J. Family Med. Prim. Care* 5 780–784. 10.4103/2249-4863.201153 28348990 PMC5353813

[B12] Bittman E. L. Kaynard A. H. Olster D. H. Robinson J. E. Yellon S. M. Karsch F. J. (1985). Pineal melatonin mediates photoperiodic control of pulsatile luteinizing hormone secretion in the ewe. *Neuroendocrinology* 40 409–418. 10.1159/000124106 3892351

[B13] Boivin D. B. Shechter A. Boudreau P. Begum E. A. Ng Ying-Kin N. M. (2016). Diurnal and circadian variation of sleep and alertness in men vs. naturally cycling women. *Proc. Natl. Acad. Sci. U.S.A.* 113 10980–10985. 10.1073/pnas.1524484113 27621470 PMC5047150

[B14] Boubekri M. Cheung I. N. Reid K. J. Wang C.-H. Zee P. C. (2014). Impact of windows and daylight exposure on overall health and sleep quality of office workers: a case-control pilot study. *J. Clin. Sleep Med.* 10 603–611. 10.5664/jcsm.3780 24932139 PMC4031400

[B15] Bubenik G. A. Brown G. M. (1997). Pinealectomy reduces melatonin levels in the serum but not in the gastrointestinal tract of rats. *Biol. Signals* 6 40–44. 10.1159/000109107 9098522

[B16] Bubenik G. A. Pang S. F. Hacker R. R. Smith P. S. (1996). Melatonin concentrations in serum and tissues of porcine gastrointestinal tract and their relationship to the intake and passage of food. *J. Pineal Res.* 21 251–256. 10.1111/j.1600-079x.1996.tb00294.x 8989725

[B17] Bugge A. Feng D. Everett L. J. Briggs E. R. Mullican S. E. Wang F. (2012). Rev-erb and Rev-erb coordinately protect the circadian clock and normal metabolic function. *Gene Dev.* 26 657–667. 10.1161/CIRCULATIONAHA.121.056076 22474260 PMC3323877

[B18] Burns G. L. Hoedt E. C. Walker M. M. Talley N. J. Keely S. (2021). Physiological mechanisms of unexplained (functional) gastrointestinal disorders. *J. Physiol.* 599 5141–5161. 10.1113/JP281620 34705270

[B19] Burr R. L. Gu H. Cain K. Djukovic D. Zhang X. Han C. (2019). Tryptophan metabolites in irritable bowel syndrome: an overnight time-course study. *J. Neurogastroenterol. Motil.* 25 551–562. 10.5056/jnm19042 31587547 PMC6786437

[B20] Cain S. W. Dennison C. F. Zeitzer J. M. Guzik A. M. Khalsa S. B. S. Santhi N. (2010). Sex differences in phase angle of entrainment and melatonin amplitude in humans. *J. Biol. Rhythms* 25 288–296. 10.1177/0748730410374943 20679498 PMC3792014

[B21] Calamini B. Santarsiero B. D. Boutin J. A. Mesecar A. D. (2008). Kinetic, thermodynamic and X-ray structural insights into the interaction of melatonin and analogues with quinone reductase 2. *Biochem. J.* 413 81–91. 10.1042/BJ20071373 18254726 PMC3265362

[B22] Camilleri M. Lasch K. Zhou W. (2012). Irritable bowel syndrome: methods, mechanisms, and pathophysiology. the confluence of increased permeability, inflammation, and pain in irritable bowel syndrome. *Am. J. Physiol. Gastrointest. Liver Physiol.* 303 G775–G785. 10.1152/ajpgi.00155.2012 22837345

[B23] Carrillo-Vico A. Calvo J. R. Abreu P. Lardone P. J. García-Mauriño S. Reiter R. J. (2004). Evidence of melatonin synthesis by human lymphocytes and its physiological significance: possible role as intracrine, autocrine, and/or paracrine substance. *FASEB J.* 18 537–539. 10.1096/fj.03-0694fje 14715696

[B24] Caruso C. C. Lusk S. L. Gillespie B. W. (2004). Relationship of work schedules to gastrointestinal diagnoses, symptoms, and medication use in auto factory workers. *Am. J. Ind. Med.* 46 586–598. 10.1002/ajim.20099 15551368

[B25] Chitkara D. K. Fortunato C. Nurko S. (2004). Prolonged monitoring of esophageal motor function in healthy children. *J. Pediatr. Gastroenterol. Nutr.* 38 192–197. 10.1097/00005176-200402000-00017 14734883

[B26] Cho H. Zhao X. Hatori M. Yu R. T. Barish G. D. Lam M. T. (2012). Regulation of circadian behaviour and metabolism by REV-ERB-α and REV-ERB-β. *Nature* 485 123–127. 22460952 10.1038/nature11048 PMC3367514

[B27] Chojnacki C. (2011). Secretion of melatonin and 6-sulfatoxymelatonin urinary excretion in functional dyspepsia. *World J. Gastroenterol.* 17:2646. 10.3748/wjg.v17.i21.2646 21677834 PMC3110928

[B28] Chojnacki C. (2013). Evaluation of enterochromaffin cells and melatonin secretion exponents in ulcerative colitis. *World J. Gastroenterol.* 19:3602. 10.3748/wjg.v19.i23.3602 23801861 PMC3691046

[B29] Chojnacki C. Walecka-Kapica E. Lokieæ K. Pawłowicz M. Winczyk K. Chojnacki J. (2013). Influence of melatonin on symptoms of irritable bowel syndrome in postmenopausal women. *Endokrynol. Pol.* 64 114–120. 23653274

[B30] Christensen M. A. Bettencourt L. Kaye L. Moturu S. T. Nguyen K. T. Olgin J. E. (2016). Direct measurements of smartphone screen-time: relationships with demographics and sleep. *PLoS One* 11:e0165331. 10.1371/journal.pone.0165331 27829040 PMC5102460

[B31] Cipolla-Neto J. Amaral F. G. Soares J. M. Gallo C. C. Furtado A. Cavaco J. E. (2021). The crosstalk between melatonin and sex steroid hormones. *Neuroendocrinology* 112 115–129. 10.1159/000516148 33774638

[B32] Colombo L. L. Chen G.-J. Lopez M. C. Watson R. R. (1992). Melatonin induced increase in gamma-interferon production by murine splenocytes. *Immunol. Lett.* 33 123–126. 10.1016/0165-2478(92)90035-m 1446916

[B33] Cummings D. E. Purnell J. Q. Frayo R. S. Schmidova K. Wisse B. E. Weigle D. S. A. (2001). Preprandial rise in plasma ghrelin levels suggests a role in meal initiation in humans. *Diabetes* 50 1714–1719. 10.2337/diabetes.50.8.1714 11473029

[B34] Damiola F. (2000). Restricted feeding uncouples circadian oscillators in peripheral tissues from the central pacemaker in the suprachiasmatic nucleus. *Gene Dev.* 14 2950–2961. 10.1101/gad.183500 11114885 PMC317100

[B35] Dawes C. (1972). Circadian rhythms in human salivary flow rate and composition. *J. Physiol.* 220 529–545. 10.1113/jphysiol.1972.sp009721 5016036 PMC1331668

[B36] Deaver J. A. Eum S. Y. Toborek M. (2018). Circadian disruption changes gut microbiome taxa and functional gene composition. *Front. Microbiol.* 9:737. 10.3389/fmicb.2018.00737 29706947 PMC5909328

[B37] Depner C. M. Melanson E. L. McHill A. W. Wright K. P. (2018). Mistimed food intake and sleep alters 24-hour time-of-day patterns of the human plasma proteome. *Proc. Natl. Acad. Sci. U.S.A.* 115 E5390–E5399. 10.1073/pnas.1714813115 29784788 PMC6003375

[B38] Drossman D. A. (1999). The functional gastrointestinal disorders and the Rome II process. *Gut* 45(Suppl. 2), ii1–ii5. 10.1136/gut.45.2008.ii1 10457038 PMC1766692

[B39] Duffy J. F. Cain S. W. Chang A. M. Phillips A. J. K. Munch M. Y. Gronfier C. (2011). Sex difference in the near-24-hour intrinsic period of the human circadian timing system. *Proc. Natl. Acad. Sci. U.S.A.* 108(Suppl._3), 15602–15608. 10.1073/pnas.1010666108 21536890 PMC3176605

[B40] Duncanson K. Burns G. Pryor J. Keely S. Talley N. J. (2021). Mechanisms of food-induced symptom induction and dietary management in functional dyspepsia. *Nutrients* 13:1109. 10.3390/nu13041109 33800668 PMC8066021

[B41] Edenberg H. J. (2007). The genetics of alcohol metabolism: role of alcohol dehydrogenase and aldehyde dehydrogenase variants. *Alcohol Res. Health* 30 5–13. 17718394 PMC3860432

[B42] Edgar D. M. Dement W. C. (1991). Regularly scheduled voluntary exercise synchronizes the mouse circadian clock. *Am. J. Physiol.* 261(4 Pt 2), R928–R933. 10.1152/ajpregu.1991.261.4.R928 1928438

[B43] Ercan F. Cetinel S. Contuk G. Cikler E. Sener G. (2004). Role of melatonin in reducing water avoidance stress-induced degeneration of the gastrointestinal mucosa. *J. Pineal Res.* 37 113–121. 10.1111/j.1600-079X.2004.00143.x 15298670

[B44] Espino J. Ortiz Á. Bejarano I. Lozano G. M. Monllor F. García J. F. (2011). Melatonin protects human spermatozoa from apoptosis via melatonin receptor- and extracellular signal-regulated kinase-mediated pathways. *Fertil. Steril.* 95 2290–2296. 10.1016/j.fertnstert.2011.03.063 21497337

[B45] Faris M. A. Kacimi S. Al-Kurd R. A. Fararjeh M. A. Bustanji Y. K. Mohammad M. K. (2012). Intermittent fasting during Ramadan attenuates proinflammatory cytokines and immune cells in healthy subjects. *Nutr. Res.* 32 947–955. 10.1016/j.nutres.2012.06.021 23244540

[B46] Fernandez B. Malde J. L. Montero A. Acuña D. (1990). Relationship between adenohypophyseal and steroid hormones and variations in serum and urinary melatonin levels during the ovarian cycle, perimenopause and menopause in healthy women. *J. Steroid Biochem.* 35 257–262. 10.1016/0022-4731(90)90282-w 2308340

[B47] Frungieri M. B. Calandra R. S. Rossi S. P. (2017). Local actions of melatonin in somatic cells of the testis. *Int. J. Mol. Sci.* 18:1170. 10.3390/ijms18061170 28561756 PMC5485994

[B48] Futagami S. Yamawaki H. Agawa S. Higuchi K. Ikeda G. Noda H. (2018). New classification Rome IV functional dyspepsia and subtypes. *Transl. Gastroenterol. Hepatol.* 3:70. 10.21037/tgh.2018.09.12 30363705 PMC6182037

[B49] Gao K. Mu C.-L. Farzi A. Zhu W.-Y. (2020). Tryptophan metabolism: a link between the gut microbiota and brain. *Adv. Nutr.* 11 709–723. 31825083 10.1093/advances/nmz127 PMC7231603

[B50] Gao T. Wang Z. Dong Y. Cao J. Chen Y. (2021). Melatonin-Mediated colonic microbiota metabolite butyrate prevents acute sleep deprivation-induced colitis in mice. *Int. J. Mol. Sci.* 22:11894. 10.3390/ijms222111894 34769321 PMC8584377

[B51] Gao T. Wang Z. Dong Y. Cao J. Lin R. Wang X. (2019). Role of melatonin in sleep deprivation-induced intestinal barrier dysfunction in mice. *J. Pineal Res.* 67:e12574. 10.1111/jpi.12574 30929267

[B52] Garcia-Mauriño S. Gonzalez-Haba M. G. Calvo J. R. Rafii-El-Idrissi M. Sanchez-Margalet V. Goberna R. (1997). Melatonin enhances IL-2, IL-6, and IFN-gamma production by human circulating CD4+ cells: a possible nuclear receptor-mediated mechanism involving T helper type 1 lymphocytes and monocytes. *J. Immunol.* 159 574–581. 9218571

[B53] García-Mauriño S. Pozo D. Carrillo-Vico A. Calvo J. R. Guerrero J. M. (1999). Melatonin activates Th1 lymphocytes by increasing IL-12 production. *Life Sci.* 65 2143–2150. 10.1016/s0024-3205(99)00479-8 10579467

[B54] Geeraerts B. Van Oudenhove L. Boesmans W. Vos R. Vanden Berghe P. Tack J. (2011). Influence of acute tryptophan depletion on gastric sensorimotor function in humans. *Am. J. Physiol. Gastrointest. Liver Physiol.* 300 G228–G235. 10.1152/ajpgi.00020.2010 20884888

[B55] Goo R. H. Moore J. G. Greenberg E. Alazraki N. P. (1987). Circadian variation in gastric emptying of meals in humans. *Gastroenterology* 93 515–518. 10.1016/0016-5085(87)90913-9 3609660

[B56] Grammaticos P. C. Doumas A. Koliakos G. (2015). Morning and night gastric emptying half-time differed more than 220% in two young healthy adults. *Hell. J. Nucl. Med.* 18 60–62. 10.1967/s002449910165 25679076

[B57] Gurel A. (2013). The role of oxidants and reactive nitrogen species in irritable bowel syndrome: a potential etiological explanation. *Med. Sci. Monit.* 19 762–766. 10.12659/MSM.889068 24029778 PMC3781198

[B58] Harlow H. J. Weekley B. L. (1986). Effect of melatonin on the force of spontaneous contractions of in vitro rat small and large intestine. *J. Pineal Res.* 3 277–284. 10.1111/j.1600-079x.1986.tb00750.x 3021949

[B59] Hastings J. W. Sweeney B. M. (1957). On the mechanism of temperature independence in a biological clock. *Proc. Natl. Acad. Sci. U.S.A.* 43 804–811. 10.1073/pnas.43.9.804 16590089 PMC534330

[B60] Höglund E. Øverli Ø. Winberg S. (2019). Tryptophan metabolic pathways and brain serotonergic activity: a comparative review. *Front. Endocrinol.* 10:158. 10.3389/fendo.2019.00158 31024440 PMC6463810

[B61] Hoogerwerf W. A. Shahinian V. B. Cornélissen G. Halberg F. Bostwick J. Timm J. (2010). Rhythmic changes in colonic motility are regulated by period genes. *Am. J. Physiol. Gastrointest. Liver Physiol.* 298 G143–G150. 10.1152/ajpgi.00402.2009 19926812 PMC2822504

[B62] Hoopes E. K. D’Agata M. N. Berube F. R. Ranadive S. M. Patterson F. Farquhar W. B. (2021). Consistency where it counts: Sleep regularity is associated with circulating white blood cell count in young adults. *Brain Behav. Immun. Health* 13:100233. 10.1016/j.bbih.2021.100233 34589748 PMC8474608

[B63] Hoskins L. C. Agustines M. McKee W. B. Boulding E. T. Kriaris M. Niedermeyer G. (1985). Mucin degradation in human colon ecosystems. Isolation and properties of fecal strains that degrade ABH blood group antigens and oligosaccharides from mucin glycoproteins. *J. Clin. Invest.* 75 944–953. 10.1172/JCI111795 3920248 PMC423632

[B64] Huether G. (1994). Melatonin synthesis in the gastrointestinal tract and the impact of nutritional factors on circulating melatonin. *Ann. N. Y. Acad. Sci.* 719 146–158. 10.1111/j.1749-6632.1994.tb56826.x 8010590

[B65] Hyun M. K. Baek Y. Lee S. (2019). Association between digestive symptoms and sleep disturbance: a cross-sectional community-based study. *BMC Gastroenterol.* 19:34. 10.1186/s12876-019-0945-9 30782128 PMC6381712

[B66] Itoh M. T. Ishizuka B. Kudo Y. Fusama S. Amemiya A. Sumi Y. (1997). Detection of melatonin and serotonin N-acetyltransferase and hydroxyindole-O-methyltransferase activities in rat ovary. *Mol. Cell. Endocrinol.* 136 7–13. 10.1016/s0303-7207(97)00206-2 9510062

[B67] Iwasaki S. Nakazawa K. Sakai J. Kometani K. Iwashita M. Yoshimura Y. (2005). Melatonin as a local regulator of human placental function. *J. Pineal Res.* 39 261–265. 10.1111/j.1600-079X.2005.00244.x 16150106

[B68] Izzo G. (2004). Inhibition of the increased 17 -estradiol-induced mast cell number by melatonin in the testis of the frog Rana esculenta, *in vivo* and *in vitro*. *J. Exp. Biol.* 207 437–441. 14691091 10.1242/jeb.00786

[B69] Johnson R. F. Moore R. Y. Morin L. P. (1988). Loss of entrainment and anatomical plasticity after lesions of the hamster retinohypothalamic tract. *Brain Res.* 460 297–313. 10.1016/0006-8993(88)90374-5 2465060

[B70] Jordan S. Tung N. Casanova-Acebes M. Chang C. Cantoni C. Zhang D. (2019). Dietary intake regulates the circulating inflammatory monocyte pool. *Cell* 178 1102–1114.e17. 10.1016/j.cell.2019.07.050 31442403 PMC7357241

[B71] Kano M. Muratsubaki T. Van Oudenhove L. Morishita J. Yoshizawa M. Kohno K. (2017). Altered brain and gut responses to corticotropin-releasing hormone (CRH) in patients with irritable bowel syndrome. *Sci. Rep.* 7:12425. 10.1038/s41598-017-09635-x 28963545 PMC5622133

[B72] Kassinen A. Krogius-Kurikka L. Mäkivuokko H. Rinttilä T. Paulin L. Corander J. (2007). The Fecal microbiota of irritable bowel syndrome patients differs significantly from that of healthy subjects. *Gastroenterology* 133 24–33. 10.1053/j.gastro.2007.04.005 17631127

[B73] Kilkens T. O. C. (2004). Acute tryptophan depletion affects brain-gut responses in irritable bowel syndrome patients and controls. *Gut* 53 1794–1800. 10.1136/gut.2004.041657 15542517 PMC1774304

[B74] Kim H. I. Jung S.-A. Choi J. Y. Kim S.-E. Jung H.-K. Shim K.-N. (2013). Impact of shiftwork on irritable bowel syndrome and functional dyspepsia. *J. Korean Med. Sci.* 28 431–437. 10.3346/jkms.2013.28.3.431 23487413 PMC3594608

[B75] Kim Y.-M. Snijders A. M. Brislawn C. J. Stratton K. G. Zink E. M. Fansler S. J. (2019). Light-Stress influences the composition of the murine gut microbiome, memory function, and plasma metabolome. *Front. Mol. Biosci.* 6:108. 10.3389/fmolb.2019.00108 31681796 PMC6813214

[B76] Kloehn I. Pillai S. B. Officer L. Klement C. Gasser P. J. Evans J. A. (2016). Sexual differentiation of circadian clock function in the adrenal gland. *Endocrinology* 157 1895–1904. 10.1210/en.2015-1968 27007073

[B77] Klupinska G. Poplawski T. Drzewoski J. Harasiuk A. Reiter R. J. Blasiak J. (2007). Therapeutic effect of melatonin in patients with functional dyspepsia. *J. Clin. Gastroenterol.* 41 270–274. 10.1097/MCG.0b013e318031457a 17426465

[B78] Knutsson A. Bøggild H. (2010). Gastrointestinal disorders among shift workers. *Scand. J. Work Environ. Health* 36 85–95. 10.5271/sjweh.2897 20101379

[B79] Koh S.-J. Kim M. Oh D. Y. Kim B. G. Lee K. L. Kim J. W. (2014). Psychosocial stress in nurses with shift work schedule is associated with functional gastrointestinal disorders. *J. Neurogastroenterol. Motil.* 20 516–522. 10.5056/jnm14034 25230903 PMC4204411

[B80] Koloski N. A. Jones M. Walker M. M. Keely S. Holtmann G. Talley J. N. (2021). Sleep disturbances in the irritable bowel syndrome and functional dyspepsia are independent of psychological distress: a population-based study of 1322 Australians. *Aliment. Pharmacol. Ther.* 54 627–636. 10.1111/apt.16500 34247414

[B81] Kostoglou-Athanassiou I. Athanassiou P. Treacher D. F. Wheeler M. J. Forsling M. L. (1998). Neurohypophysial hormone and melatonin secretion over the natural and suppressed menstrual cycle in premenopausal women. *Clin. Endocrinol.* 49 209–216. 10.1046/j.1365-2265.1998.00504.x 9828909

[B82] Kotake Y. Masayama I. (1936). The intermediary metabolism of tryptophan. XVIII. the mechanism of formation of kynurenine from tryptophan. *Z. Physiol. Chem.* 243 237–244.

[B83] Laermans J. Broers C. Beckers K. Vancleef L. Steensels S. Thijs T. (2014). Shifting the circadian rhythm of feeding in mice induces gastrointestinal, metabolic and immune alterations which are influenced by ghrelin and the core clock gene bmal1. *PLoS One* 9:e110176. 10.1371/journal.pone.0110176 25329803 PMC4199674

[B84] Lamia K. A. Papp S. J. Yu R. T. Barish G. D. Uhlenhaut N. H. Jonker J. W. (2011). Cryptochromes mediate rhythmic repression of the glucocorticoid receptor. *Nature* 480 552–556. 10.1038/nature10700 22170608 PMC3245818

[B85] Lardone P. J. Rubio A. Cerrillo I. Gómez-Corvera A. Carrillo-Vico A. Sanchez-Hidalgo M. (2010). Blocking of melatonin synthesis and MT1 receptor impairs the activation of Jurkat T cells. *Cell. Mol. Life Sci.* 67 3163–3172. 10.1007/s00018-010-0374-y 20440532 PMC11115585

[B86] Lee J.-H. Lee J. (2010). Indole as an intercellular signal in microbial communities. *FEMS Microbiol. Rev.* 34 426–444. 10.1111/j.1574-6976.2009.00204.x 20070374

[B87] Lee K. J. Kim Y. B. Kim J. H. Kwon H. C. Kim D. K. Cho S. W. (2008). The alteration of enterochromaffin cell, mast cell, and lamina propria T lymphocyte numbers in irritable bowel syndrome and its relationship with psychological factors. *J. Gastroenterol. Hepatol.* 23 1689–1694. 10.1111/j.1440-1746.2008.05574.x 19120860

[B88] Lemola S. Perkinson-Gloor N. Brand S. Dewald-Kaufmann J. F. Grob A. (2015). Adolescents’ electronic media use at night, sleep disturbance, and depressive symptoms in the smartphone age. *J. Youth Adolesc.* 44 405–418. 10.1007/s10964-014-0176-x 25204836

[B89] Lewiñski A. Zelazowski P. Sewerynek E. Zerek-Mełeñ G. Szkudliñski M. Zelazowska E. (1989). Melatonin-induced suppression of human lymphocyte natural killer activity *in vitro*. *J. Pineal Res.* 7 153–164. 2769568 10.1111/j.1600-079x.1989.tb00663.x

[B90] Liang X. Bushman F. D. Fitzgerald G. A. (2015). Rhythmicity of the intestinal microbiota is regulated by gender and the host circadian clock. *Proc. Natl. Acad. Sci. U.S.A.* 112 10479–10484. 10.1073/pnas.1501305112 26240359 PMC4547234

[B91] Liang X. Fitzgerald G. A. (2017). Timing the Microbes: the circadian rhythm of the gut microbiome. *J. Biol. Rhythms* 32 505–515. 10.1177/0748730417729066 28862076

[B92] Liu Y. Tsinoremas N. F. Johnson C. H. Lebedeva N. V. Golden S. S. Ishiura M. (1995). Circadian orchestration of gene expression in cyanobacteria. *Gene Dev.* 9 1469–1478. 10.1101/gad.9.12.1469 7601351

[B93] Lu W. Z. Gwee K. A. Moochhalla S. Ho K. Y. (2005). Melatonin improves bowel symptoms in female patients with irritable bowel syndrome: a double-blind placebo-controlled study. *Aliment. Pharmacol. Ther.* 22 927–934. 10.1111/j.1365-2036.2005.02673.x 16268966

[B94] Luboshitzky R. Lavi S. Thuma I. Lavie P. (1996). Testosterone treatment alters melatonin concentrations in male patients with gonadotropin-releasing hormone deficiency. *J. Clin. Endocrinol. Metab.* 81 770–774. 10.1210/jcem.81.2.8636302 8636302

[B95] Luna-Moreno D. Aguilar-Roblero R. Díaz-Muñoz M. (2009). Restricted feeding entrains rhythms of inflammation-related factors without promoting an acute-phase response. *Chronobiol. Int.* 26 1409–1429. 10.3109/07420520903417003 19916839

[B96] Lyra A. Rinttilä T. Nikkilä J. Krogius-Kurikka L. Kajander K. Malinen E. (2009). Diarrhoea-predominant irritable bowel syndromedistinguishable by 16S rRNA gene phylotype quantifcation. *World J. Gastroenterol.* 15:5936. 10.3748/wjg.15.5936 20014457 PMC2795180

[B97] Ma N. Zhang J. Reiter R. J. Ma X. (2020). Melatonin mediates mucosal immune cells, microbial metabolism, and rhythm crosstalk: a therapeutic target to reduce intestinal inflammation. *Med. Res. Rev.* 40 606–632. 10.1002/med.21628 31420885

[B98] Maldonado M. D. García-Moreno H. González-Yanes C. Calvo J. R. (2016). Possible involvement of the inhibition of NF-κB Factor in anti-inflammatory actions that melatonin exerts on mast cells. *J. Cell. Biochem.* 117 1926–1933. 10.1002/jcb.25491 26756719

[B99] Marquez E. Sanchez-Fidalgo S. Calvo J. R. Lastra C. A. L. D. Motilva V. (2006). Acutely administered melatonin is beneficial while chronic melatonin treatment aggravates the evolution of TNBS-induced colitis. *J. Pineal Res.* 40 48–55. 10.1111/j.1600-079X.2005.00275.x 16313498

[B100] Mazzoccoli G. Palmieri O. Corritore G. Latiano T. Bossa F. Scimeca D. (2012). Association study of a polymorphism in clock GenePERIOD3and risk of inflammatory bowel disease. *Chronobiol. Int.* 29 994–1003. 10.3109/07420528.2012.705935 22881285

[B101] Merle A. Delagrange P. Renard P. Lesieur D. Cuber J. C. Roche M. (2000). Effect of melatonin on motility pattern of small intestine in rats and its inhibition by melatonin receptor antagonist S 22153. *J. Pineal Res.* 29 116–124. 10.1034/j.1600-079x.2000.290208.x 10981825

[B102] Moayeri A. Mokhtari T. Hedayatpour A. Abbaszadeh H. A. Mohammadpour S. Ramezanikhah H. (2018). Impact of melatonin supplementation in the rat spermatogenesis subjected to forced swimming exercise. *Andrologia* 50:e12907. 10.1111/and.12907 29044638

[B103] Moja E. A. Cipolla P. Castoldi D. Tofanetti O. (1989). Dose-response decrease in plasma tryptophan and in brain tryptophan and serotonin after tryptophan-free amino acid mixtures in rats. *Life Sci.* 44 971–976. 10.1016/0024-3205(89)90497-9 2467158

[B104] Moja E. A. Stoff D. M. Gessa G. L. Castoldi D. Assereto R. Tofanetti O. (1988). Decrease in plasma tryptophan after tryptophan-free amino acid mixtures in man. *Life Sci.* 42 1551–1556. 10.1016/0024-3205(88)90013-6 3352467

[B105] Moore J. G. Halberg F. (1986). Circadian rhythm of gastric acid secretion in men with active duodenal ulcer. *Dig. Dis. Sci.* 31 1185–1191. 10.1007/BF01296516 3769701

[B106] Mrosovsky N. Edelstein K. Hastings M. H. Maywood E. S. (2001). Cycle ofperiodgene expression in a diurnal mammal (*Spermophilus tridecemlineatus*): implications for nonphotic phase shifting. *J. Biol. Rhythm* 16 471–478. 10.1177/074873001129002141 11669420

[B107] Nakajima M. (2005). Reconstitution of circadian oscillation of cyanobacterial KaiC Phosphorylation *in vitro*. *Science* 308 414–415. 10.1126/science.1108451 15831759

[B108] Natividad J. M. Agus A. Planchais J. Lamas B. Jarry A. C. Martin R. (2018). Impaired aryl hydrocarbon receptor ligand production by the gut microbiota is a key factor in metabolic syndrome. *Cell Metab.* 28 737–749.e4. 10.1016/j.cmet.2018.07.001 30057068

[B109] Nelp M. T. Zheng V. Davis K. M. Stiefel K. J. E. Groves J. T. (2019). Potent activation of indoleamine 2,3-Dioxygenase by Polysulfides. *J. Am. Chem. Soc.* 141 15288–15300. 10.1021/jacs.9b07338 31436417

[B110] Niles L. P. Wang J. Shen L. Lobb D. K. Younglai E. V. (1999). Melatonin receptor mRNA expression in human granulosa cells. *Mol. Cell. Endocrinol.* 156 107–110. 10.1016/s0303-7207(99)00135-5 10612428

[B111] Nojkov B. Rubenstein J. H. Chey W. D. Hoogerwerf W. A. (2010). The impact of rotating shift work on the prevalence of irritable bowel syndrome in nurses. *Am. J. Gastroenterol.* 105 842–847. 10.1038/ajg.2010.48 20160712 PMC2887235

[B112] Nosjean O. Ferro M. Cogé F. Beauverger P. Henlin J.-M. Lefoulon F. (2000). Identification of the Melatonin-binding SiteMT 3 as the Quinone Reductase 2. *J. Biol. Chem.* 275 31311–31317. 10913150 10.1074/jbc.M005141200

[B113] O’Connor J. C. Lawson M. A. André C. Moreau M. Lestage J. Castanon N. (2009). Lipopolysaccharide-induced depressive-like behavior is mediated by indoleamine 2,3-dioxygenase activation in mice. *Mol. Psychiatry* 14 511–522. 10.1038/sj.mp.4002148 18195714 PMC2683474

[B114] Oh-Oka K. Kono H. Ishimaru K. Miyake K. Kubota T. Ogawa H. (2014). Expressions of tight junction proteins occludin and claudin-1 are under the circadian control in the mouse large intestine: implications in intestinal permeability and susceptibility to colitis. *PLoS One* 9:e98016. 10.1371/journal.pone.0098016 24845399 PMC4028230

[B115] Pagel R. Bär F. Schröder T. Sünderhauf A. Künstner A. Ibrahim S. M. (2017). Circadian rhythm disruption impairs tissue homeostasis and exacerbates chronic inflammation in the intestine. *FASEB J.* 31 4707–4719. 10.1096/fj.201700141RR 28710114 PMC6159707

[B116] Park J. K. Huh K. C. Kwon J. G. Jung K. W. Oh J. H. Song K. H. (2021). Sleep disorders in patients with functional dyspepsia: a multicenter study from the Korean Society of Neurogastroenterology and Motility. *J. Gastroenterol. Hepatol.* 36 687–693. 10.1111/jgh.15198 32720319

[B117] Park Y. S. Kim S. H. Park J. W. Kho Y. Seok P. R. Shin J.-H. (2020). Melatonin in the colon modulates intestinal microbiota in response to stress and sleep deprivation. *Intest. Res.* 18 325–336. 10.5217/ir.2019.00093 32564539 PMC7385569

[B118] Pattison D. I. Davies M. J. Hawkins C. L. (2012). Reactions and reactivity of myeloperoxidase-derived oxidants: differential biological effects of hypochlorous and hypothiocyanous acids. *Free Radic. Res.* 46 975–995. 10.3109/10715762.2012.667566 22348603

[B119] Paulose J. K. Wright J. M. Patel A. G. Cassone V. M. (2016). Human gut bacteria are sensitive to melatonin and express endogenous circadian rhythmicity. *PLoS One* 11:e0146643. 10.1371/journal.pone.0146643 26751389 PMC4709092

[B120] Peña C. Rincon J. Pedreanez A. Viera N. Mosquera J. (2007). Chemotactic effect of melatonin on leukocytes. *J. Pineal Res.* 43 263–269. 10.1111/j.1600-079X.2007.00471.x 17803523

[B121] Pentney P. T. Bubenik G. A. (1995). Melatonin reduces the severity of dextran-induced colitis in mice. *J. Pineal Res.* 19 31–39. 10.1111/j.1600-079x.1995.tb00168.x 8609593

[B122] Pioli C. Caroleo M. C. Nistico G. Doriac G. (1993). Melatonin increases antigen presentation and amplifies specific and non specific signals for T-cell proliferation. *Int. J. Immunopharmacol.* 15 463–468. 10.1016/0192-0561(93)90060-c 8365822

[B123] Plautz J. D. Kaneko M. Hall J. C. Kay S. A. (1997). Independent photoreceptive circadian clocks throughoutdrosophila. *Science* 278 1632–1635. 9374465 10.1126/science.278.5343.1632

[B124] Preitner N. Damiola F. Luis Lopez M. Zakany J. Duboule D. Albrecht U. (2002). The orphan nuclear receptor REV-ERBα controls circadian transcription within the positive limb of the mammalian circadian oscillator. *Cell* 110 251–260. 10.1016/s0092-8674(02)00825-5 12150932

[B125] Preuss F. Tang Y. Laposky A. D. Arble D. Keshavarzian A. Turek F. W. (2008). Adverse effects of chronic circadian desynchronization in animals in a “challenging” environment. *Am. J. Physiol. Regul. Integr. Comp. Physiol.* 295 R2034–R2040. 10.1152/ajpregu.00118.2008 18843092 PMC2685296

[B126] Pridmore R. D. Berger B. Desiere F. Vilanova D. Barretto C. Pittet A. C. (2004). The genome sequence of the probiotic intestinal bacterium Lactobacillus johnsonii NCC 533. *Proc. Natl. Acad. Sci.U.S.A.* 101 2512–2517. 10.1073/pnas.0307327101 14983040 PMC356981

[B127] Radwan P. Skrzydlo-Radomanska B. Radwan-Kwiatek K. Burak-Czapiuk B. Strzemecka J. (2009). Is melatonin involved in the irritable bowel syndrome? *J. Physiol. Pharmacol.* 60(Suppl. 3), 67–70. 19996484

[B128] Raghavendra V. Singh V. Kulkarni S. K. Agrewala J. N. (2001). Melatonin enhances Th2 cell mediated immune responses: lack of sensitivity to reversal by naltrexone or benzodiazepine receptor antagonists. *Mol. Cell. Biochem.* 221 57–62. 10.1023/a:1010968611716 11506187

[B129] Raikhlin N. T. Kvetnoy I. M. (1976). Melatonin and enterochromaffine cells. *Acta Histochem.* 55 19–24. 10.1016/S0065-1281(76)80092-X 818867

[B130] Rajiliæ-Stojanoviæ M. Biagi E. Heilig H. G. H. J. Kajander K. Kekkonen R. A. Tims S. (2011). Global and deep molecular analysis of microbiota signatures in fecal samples from patients with irritable bowel syndrome. *Gastroenterology* 141 1792–1801. 10.1053/j.gastro.2011.07.043 21820992

[B131] Redman J. Armstrong S. Ng K. (1983). Free-running activity rhythms in the rat: entrainment by melatonin. *Science* 219 1089–1091. 10.1126/science.6823571 6823571

[B132] Roenneberg T. Wirz-Justice A. Merrow M. (2003). life between clocks: daily temporal patterns of human chronotypes. *J. Biol. Rhythm* 18 80–90. 10.1177/0748730402239679 12568247

[B133] Rosselot A. E. Hong C. I. Moore S. R. (2016). Rhythm and bugs. *Curr. Opin. Gastroenterol.* 32 7–11. 10.1097/mog.0000000000000227 26628099 PMC4721637

[B134] Saeed A. M. Galal I. H. (2017). Irritable bowel syndrome in obstructive sleep apnea: a preliminary Egyptian study. *Egypt. J. Bronchol.* 11 379–385. 10.4103/1687-8426.217636

[B135] Saha L. Malhotra S. Rana S. Bhasin D. Pandhi P. (2007). A preliminary study of melatonin in irritable bowel syndrome. *J. Clin. Gastroenterol.* 41 29–32. 10.1097/MCG.0b013e31802df84c 17198061

[B136] Sakaguchi K. Itoh M. T. Takahashi N. Tarumi W. Ishizuka B. (2013). The rat oocyte synthesises melatonin. *Reprod. Fertil. Dev.* 25 674–682. 10.1071/RD12091 22951050

[B137] Saulnier D. M. Riehle K. Mistretta T. A. Diaz M. A. Mandal D. Raza S. (2011). Gastrointestinal microbiome signatures of pediatric patients with irritable bowel syndrome. *Gastroenterology* 141 1782–1791. 10.1053/j.gastro.2011.06.072 21741921 PMC3417828

[B138] Schiavo-Cardozo D. Lima M. M. O. Pareja J. C. Geloneze B. (2013). Appetite-regulating hormones from the upper gut: disrupted control of xenin and ghrelin in night workers. *Clin. Endocrinol.* 79 807–811. 10.1111/cen.12114 23199168

[B139] Scott S. A. Fu J. Chang P. V. (2020). Microbial tryptophan metabolites regulate gut barrier function via the aryl hydrocarbon receptor. *Proc. Natl. Acad. Sci.U.S.A.* 117 19376–19387. 10.1073/pnas.2000047117 32719140 PMC7431026

[B140] Sen S. Dumont S. Sage-Ciocca D. Reibel S. De Goede P. Kalsbeek A. (2018). Expression of the clock gene Rev-erbα in the brain controls the circadian organisation of food intake and locomotor activity, but not daily variations of energy metabolism. *J. Neuroendocrinol.* 30:e12557. 10.1111/jne.12557 29150901

[B141] Shaji A. V. Kulkarni S. K. Agrewala J. N. (1998). Regulation of secretion of IL-4 and IgG1 isotype by melatonin-stimulated ovalbumin-specific T cells. *Clin. Exp. Immunol.* 111 181–185. 9472679 10.1046/j.1365-2249.1998.00493.x PMC1904848

[B142] Sharma S. Haldar C. (2006). Melatonin prevents X-ray irradiation induced oxidative damagein peripheral blood and spleen of the seasonally breeding rodent, Funambulus pennantiduring reproductively active phase. *Int. J. Radiat. Biol.* 82 411–419. 10.1080/09553000600774105 16846976

[B143] Shearman L. P. (2000). Interacting molecular loops in the mammalian circadian clock. *Science* 288 1013–1019. 10.1126/science.288.5468.1013 10807566

[B144] Shivaji U. N. Ford A. C. (2014). Prevalence of functional gastrointestinal disorders among consecutive new patient referrals to a gastroenterology clinic. *Frontline Gastroenterol.* 5:266–271. 10.1136/flgastro-2013-100426 28839783 PMC5369735

[B145] Söderquist F. Hellström P. M. Cunningham J. L. (2015). Human gastroenteropancreatic expression of melatonin and its receptors MT1 and MT2. *PLoS One* 10:e0120195. 10.1371/journal.pone.0120195 25822611 PMC4378860

[B146] Song G. H. (2005). Melatonin improves abdominal pain in irritable bowel syndrome patients who have sleep disturbances: a randomised, double blind, placebo controlled study. *Gut* 54 1402–1407. 10.1136/gut.2004.062034 15914575 PMC1774717

[B147] Sperber A. D. Bangdiwala S. I. Drossman D. A. Ghoshal U. C. Simren M. Tack J. (2021). Worldwide prevalence and burden of functional gastrointestinal disorders, results of rome foundation global study. *Gastroenterology* 160:99. 10.1053/j.gastro.2020.04.014 32294476

[B148] Stanghellini V. Chan F. K. L. Hasler W. L. Malagelada J. R. Suzuki H. Tack J. (2016). Gastroduodenal Disorders. *Gastroenterology* 150 1380–1392. 27147122 10.1053/j.gastro.2016.02.011

[B149] Stebelová K. Anttila K. Mänttäri S. Saarela S. Zeman M. (2010). Immunohistochemical definition of MT2 receptors and melatonin in the gastrointestinal tissues of rat. *Acta Histochem.* 112 26–33. 19004484 10.1016/j.acthis.2008.03.004

[B150] Stevenson N. R. Sitren H. S. Furuya S. (1980). Circadian rhythmicity in several small intestinal functions is independent of use of the intestine. *Am. J. Physiol.* 238 203–207. 10.1152/ajpgi.1980.238.3.G203 6102847

[B151] Storr M. Koppitz P. Sibaev A. Saur D. Kurjak M. Franck H. (2002). Melatonin reduces non-adrenergic, non-cholinergic relaxant neurotransmission by inhibition of nitric oxide synthase activity in the gastrointestinal tract of rodents *in vitro*. *J. Pineal Res.* 33 101–108. 12153444 10.1034/j.1600-079x.2002.02909.x

[B152] Storr M. Schusdziarra V. Allescher H. D. (2000). Inhibition of small conductance K+ -channels attenuated melatonin-induced relaxation of serotonin-contracted rat gastric fundus. *Can. J. Physiol. Pharmacol.* 78 799–806. 11077980

[B153] Summa K. C. Voigt R. M. Forsyth C. B. Shaikh M. Cavanaugh K. Tang Y. (2013). Disruption of the circadian clock in mice increases intestinal permeability and promotes alcohol-induced hepatic pathology and inflammation. *PLoS One* 8:e67102. 10.1371/journal.pone.0067102 23825629 PMC3688973

[B154] Tanaka Y. Kanazawa M. Kano M. Morishita J. Hamaguchi T. Van Oudenhove L. (2016). Differential activation in amygdala and plasma noradrenaline during colorectal distention by administration of corticotropin-releasing hormone between healthy individuals and patients with irritable bowel syndrome. *PLoS One* 11:e0157347. 10.1371/journal.pone.0157347 27448273 PMC4957789

[B155] Tang Y. Preuss F. Turek F. W. Jakate S. Keshavarzian A. (2009). Sleep deprivation worsens inflammation and delays recovery in a mouse model of colitis. *Sleep Med.* 10 597–603. 19403332 10.1016/j.sleep.2008.12.009 PMC3509796

[B156] Taylor D. J. Mallory L. J. Lichstein K. L. Durrence H. H. Riedel B. W. Bush A. J. (2007). Comorbidity of chronic insomnia with medical problems. *Sleep* 30 213–218. 17326547 10.1093/sleep/30.2.213

[B157] Thaiss C. A. Levy M. Korem T. Dohnalová L. Shapiro H. Jaitin D. A. (2016). Microbiota diurnal rhythmicity programs host transcriptome oscillations. *Cell* 167 1495–1510.e12. 27912059 10.1016/j.cell.2016.11.003

[B158] Thaiss C. A. Zeevi D. Levy M. Zilberman-Schapira G. Suez J. Tengeler A. C. (2014). Transkingdom control of microbiota diurnal oscillations promotes metabolic homeostasis. *Cell* 159 514–529. 25417104 10.1016/j.cell.2014.09.048

[B159] Tran L. Jochum S. B. Shaikh M. Wilber S. Zhang L. Hayden D. M. (2021). Circadian misalignment by environmental light/dark shifting causes circadian disruption in colon. *PLoS One* 16:e0251604. 10.1371/journal.pone.0251604 34086699 PMC8177509

[B160] Trefely S. Lovell C. D. Snyder N. W. Wellen K. E. (2020). Compartmentalised acyl-CoA metabolism and roles in chromatin regulation. *Mol. Metab.* 38:100941. 10.1016/j.molmet.2020.01.005 PMC7300382 32199817

[B161] Tu Q. Heitkemper M. M. Jarrett M. E. Buchanan D. T. (2017). Sleep disturbances in irritable bowel syndrome: a systematic review. *Neurogastroenterol. Motil.* 29:e12946. 10.1111/nmo.12946 27683238

[B162] Túnez I. Muñoz M. C. Medina F. J. Salcedo M. Feijóo M. Montilla P. (2007). Comparison of melatonin, vitamin E and L-carnitine in the treatment of neuro- and hepatotoxicity induced by thioacetamide. *Cell Biochem. Funct.* 25 119–127. 16245358 10.1002/cbf.1276

[B163] Vanheel H. Vicario M. Vanuytsel T. Van Oudenhove L. Martinez C. Keita Å. V. (2014). Impaired duodenal mucosal integrity and low-grade inflammation in functional dyspepsia. *Gut* 63 262–271. 23474421 10.1136/gutjnl-2012-303857

[B164] Voigt R. M. Forsyth C. B. Keshavarzian A. (2019). Circadian rhythms: a regulator of gastrointestinal health and dysfunction. *Expert Rev. Gastroenterol. Hepatol.* 13 411–424. 30874451 10.1080/17474124.2019.1595588 PMC6533073

[B165] Walker M. M. Talley N. J. Prabhakar M. Pennaneac’H C. J. Aro P. Ronkainen J. (2009). Duodenal mastocytosis, eosinophilia and intraepithelial lymphocytosis as possible disease markers in the irritable bowel syndrome and functional dyspepsia. *Aliment. Pharmacol. Ther.* 29 765–773. 19183150 10.1111/j.1365-2036.2009.03937.x PMC4070654

[B166] Wams E. J. Woelders T. Marring I. Van Rosmalen L. Beersma D. G. M. Gordijn M. C. M. (2017). Linking light exposure and subsequent sleep: a field polysomnography study in humans. *Sleep* 40:zsx165. 10.1093/sleep/zsx165 PMC5806586 29040758

[B167] Wang C. Zhang Z.-M. Xu C.-X. Tischkau S. (2014). Interplay between dioxin-mediated signaling and circadian clock: a possible determinant in metabolic homeostasis. *Int. J. Mol. Sci.* 15 11700–11712. 24987953 10.3390/ijms150711700 PMC4139808

[B168] Wang L. Christophersen C. T. Sorich M. J. Gerber J. P. Angley M. T. Conlon M. A. (2013). Increased abundance of *Sutterella* spp. and Ruminococcus torques in feces of children with autism spectrum disorder. *Mol. Autism* 4:42. 10.1186/2040-2392-4-42 PMC3828002 24188502

[B169] Wang X. S. Armstrong M. E. G. Cairns B. J. Key T. J. Travis R. C. (2011). Shift work and chronic disease: the epidemiological evidence. *Occup. Med.* 61 78–89. 10.1093/occmed/kqr001 PMC3045028 21355031

[B170] Wehrens S. M. T. Christou S. Isherwood C. Middleton B. Gibbs M. A. Archer S. N. (2017). Meal timing regulates the human circadian system. *Curr. Biol.* 27:1768. 10.1016/j.cub.2017.04.059 PMC5483233 28578930

[B171] Wei L. Yue F. Xing L. Wu S. Shi Y. Li J. (1975). Constant light exposure alters gut microbiota and promotes the progression of steatohepatitis in high fat diet rats. *Front. Microbiol.* 11:1975. 10.3389/fmicb.2020.01975 32973715 PMC7472380

[B172] Willerford D. M. Chen J. Ferry J. A. Davidson L. Ma A. Alt F. W. (1995). Interleukin-2 receptor alpha chain regulates the size and content of the peripheral lymphoid compartment. *Immunity* 3 521–530. 7584142 10.1016/1074-7613(95)90180-9

[B173] Williams B. B. Van Benschoten A. H. Cimermancic P. Donia M. S. Zimmermann M. Taketani M. (2014). Discovery and characterization of gut microbiota decarboxylases that can produce the neurotransmitter tryptamine. *Cell Host Microbe* 16 495–503. 25263219 10.1016/j.chom.2014.09.001 PMC4260654

[B174] Wisniewska-Jarosinska M. Chojnacki J. Konturek S. Brzozowski T. Smigielski J. Chojnacki C. (2010). Evaluation of urinary 6-hydroxymelatonin sulphate excretion in women at different age with irritable bowel syndrome. *J. Physiol. Pharmacol.* 61 295–300. 20610859

[B175] Woo M. M. Tai C. J. Kang S. K. Nathwani P. S. Pang S. F. Leung P. C. (2001). Direct action of melatonin in human granulosa-luteal cells. *J. Clin. Endocrinol. Metab.* 86 4789–4797. 11600542 10.1210/jcem.86.10.7912

[B176] Wright K. P. Jr. McHill A. W. Birks B. R. Griffin B. R. Rusterholz T. Chinoy E. D. (2013). Entrainment of the human circadian clock to the natural light-dark cycle. *Curr. Biol.* 23 1554–1558. 23910656 10.1016/j.cub.2013.06.039 PMC4020279

[B177] Yamaguchi M. Kotani K. Tsuzaki K. Takagi A. Motokubota N. Komai N. (2015). Circadian rhythm genes CLOCK and PER3 polymorphisms and morning gastric motility in humans. *PLoS One* 10:e0120009. 10.1371/journal.pone.0120009 25775462 PMC4388463

[B178] Yamawaki H. Futagami S. Shimpuku M. Sato H. Wakabayashi T. Maruki Y. (2014). Impact of sleep disorders, quality of life and gastric emptying in distinct subtypes of functional dyspepsia in Japan. *J. Neurogastroenterol. Motil.* 20 104–112. 24466451 10.5056/jnm.2014.20.1.104 PMC3895596

[B179] Yang P. L. Burr R. L. Buchanan D. T. Pike K. C. Kamp K. J. Heitkemper M. M. (2020). Indirect effect of sleep on abdominal pain through daytime dysfunction in adults with irritable bowel syndrome. *J. Clin. Sleep Med.* 16 1701–1710. 32620184 10.5664/jcsm.8658 PMC7954013

[B180] Yang W. C. Tang K. Q. Fu C. Z. Riaz H. Zhang Q. Zan L. S. (2014). Melatonin regulates the development and function of bovine Sertoli cells via its receptors MT1 and MT2. *Anim. Reprod. Sci.* 147 10–16. 24768045 10.1016/j.anireprosci.2014.03.017

[B181] Yano J. M. Yu K. Donaldson G. P. Shastri G. G. Ann P. Ma L. (2015). Indigenous bacteria from the gut microbiota regulate host serotonin biosynthesis. *Cell* 161 264–276. 25860609 10.1016/j.cell.2015.02.047 PMC4393509

[B182] Yoshida D. Aoki N. Tanaka M. Aoyama S. Shibata S. (2015). The circadian clock controls fluctuations of colonic cell proliferation during the light/dark cycle via feeding behavior in mice. *Chronobiol. Int.* 32 1145–1155. 26376208 10.3109/07420528.2015.1065415

[B183] Zarrinpar A. Chaix A. Yooseph S. Panda S. (2014). Diet and feeding pattern affect the diurnal dynamics of the gut Microbiome. *Cell Metab.* 20 1006–1017. 25470548 10.1016/j.cmet.2014.11.008 PMC4255146

[B184] Zheng L. Seon Y. J. McHugh J. Papagerakis S. Papagerakis P. (2012). Clock genes show circadian rhythms in salivary glands. *J. Dent. Res.* 91 783–788. 22699207 10.1177/0022034512451450 PMC3398790

[B185] Zhong L. Shanahan E. R. Raj A. Koloski N. A. Fletcher L. Morrison M. (2017). Dyspepsia and the microbiome: time to focus on the small intestine. *Gut* 66 1168–1169. 27489239 10.1136/gutjnl-2016-312574

[B186] Zybach K. (2016). Therapeutic effect of melatonin on pediatric functional dyspepsia: a pilot study. *World J. Gastrointest. Pharmacol. Ther.* 7 156–161. 26855822 10.4292/wjgpt.v7.i1.156 PMC4734949

